# Traditional Chinese Herbal Medicines for Treating HIV Infections and AIDS

**DOI:** 10.1155/2012/950757

**Published:** 2012-12-27

**Authors:** Wen Zou, Ying Liu, Jian Wang, Hongjuan Li, Xing Liao

**Affiliations:** ^1^Center of AIDS Treatment with Traditional Chinese Medicine, China Academy of Chinese Medical Sciences, 16 Dongzhimennei South Street, Dongcheng District, Beijing 100700, China; ^2^Department of Diagnosis with Chinese Medicine, University of Traditional Chinese Medicine, Beijing 100029, China; ^3^Institute of Basic Research in Clinical Medicine, China Academy of Chinese Medical Sciences, Beijing 100700, China

## Abstract

To assess the effects of TCHM on patients with HIV infection and AIDS, we reviewed eleven randomized placebo-controlled trials involving 998 patients. Due to the limited number of RCTs for included trials and the small sample size of each study, we are not able to draw firm conclusions concerning TCHM therapy in treating patients with HIV infection and AIDS. However, some high-quality clinical studies do exist. Studies of diarrhea and oral candidiasis, which are challenging symptoms of AIDS, were demonstrated to have positive effects. Study of peripheral leukocytes, which are a side effect of antiretroviral drugs, suggested that an integrated treatment approach may be of benefit. The overall methodological quality of the trials was adequate; however, randomization methods should be clearly described and fully reported in these trials according to the Consolidated Standards of Reporting Trials (CONSORT).

## 1. Introduction

Although human immunodeficiency virus (HIV) infection was first reported in China in 1985, the magnitude of its spread was not evident until the epidemic among former plasma donors across central China was realized. Poor, rural farmers sold plasma to unscrupulous collectors under unsanitary conditions during the early to mid-1990s, resulting in untold numbers of infections [1]. The Chinese government initiated the China Comprehensive acquired immuno-deficiency syndrome (AIDS) Response program, which is also called China National Free Antiretroviral Treatment Programme (NFATP), to provide free HIV treatment in 127 counties with former plasma donors across central China. This program began as a pilot—treating 100 patients in 2002—but was rapidly scaled up to cover 82,540 patients to the rest of the country by the end of 2009 [2]. 

To date, an estimated 740,000 people in China are infected with HIV, most of whom are injecting drug users (IDUs), female sex workers, men who have sex with men, former plasma donors, or blood transfusion recipients. As of Dec 31, 2009, 323,252 people were reported as having HIV in China [3]. People living with HIV and AIDS have become a significant health issue in China, and an increasing number of HIV-infected individuals are in need of care.

The availability of highly active antiretroviral therapy (HAART) has markedly improved the survival rate and quality of life in patients infected with HIV, data from the NFATP show virological suppression, increased CD4+ cell counts, and a pronounced decrease in mortality in patients who have received treatment [4]. At present, however, there is still no cure for HIV. The use of antiretroviral drugs has been associated with several toxicities that limit their success. Some acute and chronic toxicities associated with these drugs include hypersensitivity reactions, neurotoxicity, nephropathy, liver damage, and the appearance of body fat redistribution syndrome and the different metabolic alterations that accompany it. The persistence of prolonged HIV reservoirs in patients on effective antiretroviral therapy is the main hurdle to HIV eradication [5].

Three types of treatment systems are practiced in Chinese society: (a) HAART offered by health care professionals in clinics and hospitals; (b) Buyao, which is over-the-counter popular medicine and includes teas, soups, tablets, herbal preparations, and tonics, which are similar to herb supplements used in some Western countries; and (c) traditional chinese medicine (TCM), provided by trained Chinese herbalists, which incorporates a wide range of theories, therapies, and practices. Many Chinese people use all three types of treatment simultaneously. Generally, people with HIV infection use TCHM for four main reasons: to enhance their immune function, to treat symptoms, to improve their quality of life (QoL), and to reduce side effects related to medications [6]. Comprehensive TCM intervention started from 2004, National Free TCM HIV/AIDS Treatment Program had been launched by The State Administrative bureau of Traditional Chinese Medicine, and quickly scaled up from 5 provinces (Henan, Hebei, Anhui, Hubei, Guangdong) to 19 provinces, autonomous regions, and municipalities in China, 9267 cases have been treated with TCM accumulatively by 2009, retrospective data analysis suggested promising effect in promoting CD4 + cell [7].

When HAART is the dominant method of treatment, however, its use is complemented by the presence of complementary and alternative medicine (CAM) [8]. The majority of people living with HIV/AIDS are using complementary medicine [9], in China and South Africa these treatments are used as primary treatments [10]. Commonly, CAM includes a wide range of practices that do not fit within the dominant allopathic model of health care [11], including but not limited to herbalism, traditional chinese herbal medicine (TCHM), acupuncture, and diet-based therapies. TCHM has been used in Chinese society for more than 5,000 years. In the TCHM approach, the body is recognized and treated as a whole entity, and diseases are identified as conditions caused by internal imbalances. The role of doctors is to identify imbalances and then correct them; the body is then expected to be able to heal itself [12]. TCHM, among the most widespread of complementary therapeutic modalities, are defined in this review as herbal Chinese medicine products derived from plants or parts of plants used for the treatment of HIV/AIDS.

The objective of this paper is to assess the beneficial and harmful effects of TCHM on patients with HIV infection and AIDS compared with no intervention, placebo, or antiretroviral drug.

## 2. Methods

### 2.1. Inclusion Criteria

Randomised controlled trials (RCTs) of TCHM in people with HIV infection, HIV-related disease, or AIDS are included irrespective of publication status or language. Observational studies and case series were excluded. TCHM is defined in this paper as herbal Chinese medicine products derived from plants or parts of plants used for the treatment of HIV/AIDS. Controlled intervention can be no treatment, placebo, or antiretrovirals (monotherapy and combination therapies, including HAART).

### 2.2. Search Strategy for the Identification of Studies

The following electronic databases were searched (between January 1982 and December 2011):

- Cochrane HIV/AIDS Group Trials Register, CENTRAL database, the Cochrane Complementary Medicine Field;
- MEDLINE, EMBASE, LILACS, Science Citation Index (SCI), China Network Knowledge Infrastructure (CNKI), and the Chinese Biomedical CD-ROM Database, Traditional Chinese Medical Literature Analysis and Retrieval System (TCMLARS).

The search terms included HIV, acquired-immunodeficiency-syndrome (AIDS), Chinese-medicine, medicine-Chinese-traditional, medicine-Chinese-herb, herbs, Chinese-herbs.

Chinese Journal of Infectious Diseases, Chinese Journal of Dermatovenereology, Journal for China AIDS/STD Prevention and Control, Chinese Journal of Integrated Traditional and Western Medicine, Research of Traditional Chinese Medicine, and Journal of Traditional Chinese Medicine were handsearched from the first publication date onwards to December 2011. Conference proceedings in Chinese were also handsearched.

Manufacturers of herbal preparations and experts in relevant fields were contacted for potential trials. The bibliographies of identified trials and review articles were checked in order to find randomised trials not identified by the electronic searches or handsearches.

### 2.3. Data Extraction, Quality, and Risk of Bias Assessment

All articles were read, and data were extracted from the articles based on predefined selection criteria by two independent reviewers. To evaluate the methodological quality of the RCTs, the risk of bias was determined using the Cochrane classification for eight criteria: random sequence generation, allocation concealment, patient blinding, assessor blinding, reporting of dropout or withdrawal, intention-to-treat analysis, selective outcome reporting, and other potential biases [24].

## 3. Results

### 3.1. Study Description

**Figure 1:**
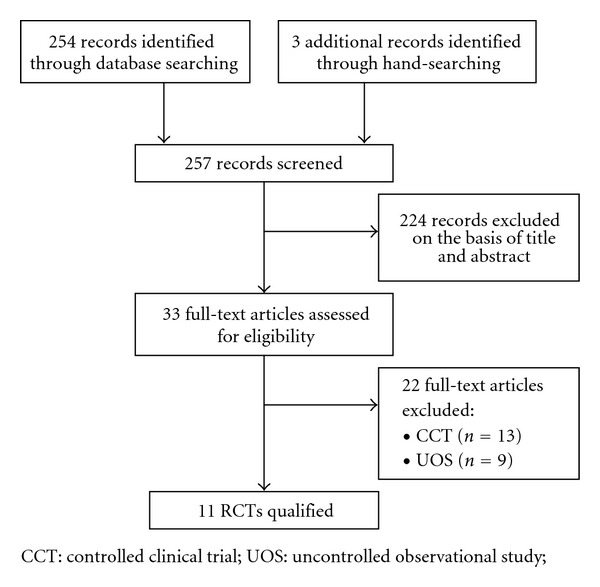
Flow diagram of literature search.

We screened 257 relevant articles, and 224 were excluded, leaving us with 33 full-text eligible articles. Of these, 22 more were excluded. The remaining 11 RCTs met our inclusion criteria (Figure 1).

Key data from these studies are summarized in Tables 1 and 2 [13–23]. Eleven different kinds of Chinese medicines in a total of 998 patients with HIV infection or AIDS were tested. A placebo procedure was employed in all 11 trials. All of the included trials adopted a two-arm parallel group design [13–23].

### 3.2. Risk of Bias

The risk of bias in the studies was variable. Nine RCTs had an adequate method for random sequence generation [13, 15–18, 20–23], whereas the remaining 2 RCTs did not describe [14, 19]. Allocation concealments were adequately performed in 10 RCTs [13–19, 21–23]. Patient and assessor blinding was reported in 10 of the RCTs [13–19, 21–23], whereas one RCT employed patient blinding only [22]. Reasons for dropouts and withdrawals were fully described in 11 trials [13–23]. Only four studies employed the ITT method [13, 17, 21, 23], but the remaining studies had missing outcome data balanced in numbers across intervention groups, with similar reasons for missing data across groups, except one study [14]. All of the included RCTs had a low risk of bias in selective outcome reporting. The sample size ranged from 30–176 patients. Overall, the methodological quality of the trials was adequate.

### 3.3. Efficacy and Safety

Due to the limited number of trials identified and the variation of participants and herbal preparations, meta-analysis and the prespecified subgroup or sensitivity analyses were not performed.

#### 3.3.1. IGM-1

A randomized trial tested a Chinese herbal formulation (IGM-1) composed of 31 Chinese herbs (Table 1) in 30 HIV-infected adults with symptoms and decreased CD4+ cell count (200–499/mm^3^) for treatment of HIV-related symptoms for duration of 12 weeks [13]. The study found a significant better effect in improvement of health-related QoL in terms of life satisfaction and symptoms than placebo. The number of symptoms was reduced in patients receiving herbs, but not in those receiving placebo. There were no statistically significant differences in overall health perception, symptom severity, CD4 counts, anxiety, or depression between groups. No adverse events were reported among participants. However, the above results need to be accounted for with care due to the small sample in the trial.

#### 3.3.2. “35-Herb”

Interestingly, three years after the above trial was published, the same investigator who prescribed IGM-1 prescribed another Chinese herbal formulation that was tested in a trial in Switzerland [17]. The formulation was composed of 35 Chinese herbs containing most of the herbs listed in IGM-1 (Table 1). A trial tested the Chinese herbal formulation in 68 HIV-infected adults with decreased CD4+ cell count (less than 500/mm^3^) for a treatment period of six months [17]. The participants were randomized to receive “35-herb” (*n* = 34) or placebo (*n* = 34). Over 70% of the patients had received previous antiretroviral therapy, the two groups were comparable regarding sociodemographic characteristics, previous antiretroviral use, viral load, CD4+ cell counts, and other clinical laboratory tests at entry. A total of 53 (78%) patients completed treatment for 6 months, including 24 in the herb group and 29 in the placebo group. Analyses were based on complete data and on intention-to-treat principle in the trial report. After six months, there was no significant difference in CD4+ cell counts, viral load, new AIDS-defining events, number of reported symptoms, psychosocial measurements or QoL between two groups.

The total number of reported adverse events was 46 in the herb group and 20 in the placebo group, and included diarrhea, increased number of daily bowel movements, abdominal pain, constipation, flatulence, and nausea. Hematological or serum chemistry laboratory values showed no evidence of toxicity from the study herbs. Two patients in the herb group died during the study period and causes of death were believed to be due to severe immunodeficiency and pre-enrolment history of severe opportunistic complications, but not related to the study drugs.

#### 3.3.3. Compound SH

Compound SH containing five herbs (Table 1) were combined with zidovudine and zalcitabine in the treatment of 60 HIV-infected Thai patients in a randomized trial [14]. The herbal formula was made from more than 1000 chinese herbs from 120 plant families by Kunming Institute of Botany of the Chinese Academy of Science. The trial found that adding SH herbs to the two nucleoside reverse transcriptase inhibitors has a greater antiviral activity than antiretrovirals only. However, the data analyses were based on participants, who had completed the trial, 22 subjects who lost followup or withdrawal due to adverse events were excluded, and the above benefits need to be accounted for with care.

#### 3.3.4. Qiankunning

Qiankunning (Table 1) is a Chinese herb preparation extracted from 14 herbs. A randomized, double blind placebo controlled trial was conducted in 2003 in China [15], 36 adults with HIV infection or AIDS were randomized to receive Qiankunning (*n* = 18) or placebo (*n* = 18). Patients were comparable regarding age, body weight, average duration of drug abuse, and pre trial HIV RNA levels. No intention to treat analyses were applied, the data analyses were based on participants who had completed the trial. Significant decrease in HIV RNA levels was found in herb group than placebo after the end of treatment for 7 months. In this trial, the use of herbs was related to stomach discomfort and diarrhea. No adverse effects were reported from the placebo group. There were no serious adverse events observed.

#### 3.3.5. Zhongyan-4

Chinese herbal medicine zhongyan-4 (ZY-4) (Table 1) is prepared by the Chinese Academy of Chinese Medical Sciences in Beijing, China. A randomized, double blind placebo controlled trial enrolled 72 patients with HIV infection or AIDS (36 with herbs and 36 with placebo) [16]. CD4+ cell counts in the ZY-4 group were increased by 7.7 ± 150.96 cells/mm^3^, while in the placebo group the CD4+ cell counts were decreased by 27.33 ± 85.28 cells/mm^3^ after treatment for 6 months (*P* > 0.05). A total of 15 out of 30 patients (6 dropped out) in the ZY-4 group had their CD4 count increased compared with 8 out of 33 patients (3 dropped out) in the placebo group (*P* < 0.05). The study concludes that ZY-4 is effective in enhancing immunity function based on CD4+ cell counts. However, this study showed no significant difference in body weight or viral load after treatment between ZY-4 and placebo.

#### 3.3.6. Aining Granule

The Chinese herbal medicine Aining Granule (AG) (Table 1) was tested in 100 patients compared with placebo in a double blind trial. Participants were randomized into two groups [18], AG group (*n* = 50) received AG+HAART (d4T+ddI+NVP) and Placebo group (*n* = 50) received placebo + HAART (d4T+ddI+NVP). CD4+ cell counts in the AG group were decreased by 87.65 ± 107.98 cells/mm^3^, while in the placebo group the CD4+ cell counts were decreased by 156.51 ± 157.04 cells/mm^3^ after treatment for 11 months (*P* < 0.05). Significant improvement of symptoms such as fatigue, anorexia, nausea, diarrhea, skin rash was found in AG group. The results showed that patients receiving Chinese herb AG had a lower risk for the decrease of CD4+ cell counts. However, this study showed no significant difference between two groups in viral load after treatment.

#### 3.3.7. Xiaomi Granule

A randomized two arms positive-drug controlled open label trial was conducted in 2009 in china, in which 80 AIDS participants with oral candidiasis were included in the Xiaomi Granule (Table 1) plus Nystatin group (*n* = 40) and Nystatin group (*n* = 40) [19]. After treatment for 2 weeks, significant improvement of symptoms of oral candidiasis was found in herb group. No adverse event was found. Xiaomi Granule is a Chinese herb preparation developed from a prescription in classic Chinese medicine ancient book “jin kui yao lve”. There is no description of CD4+ cell counts and viral load in the paper available.

#### 3.3.8. Jingyuankang Capsule

In a double-blind, double-analogue trial, 116 participants with HIV infection and peripheral leucopenia were randomized to receive Jingyuankang Capsule (JC) (Table 1) plus AZT, ddI, NVP, and analogue Leucogen Tablets (*n* = 58) or Leucogen Tablets plus AZT, ddI, NVP and analogue JC (*n* = 58) for 6 months [20]. The application of JC showed significant increase of peripheral leukocytes in herb group. CD4+ cell count outcome was not reported. There were no significant differences between the groups regarding adverse effect in the trial report.

#### 3.3.9. Xielikang Capsule

A randomized, double-blind, double dummy and controlled clinical trial was conducted between 2009 and 2011 in china, in which 158 AIDS-related chronic diarrhea patients were randomized into Xielikang Capsule (XC) (Table 1) plus loperamide analogue group (*n* = 106) and loperamide capsule plus XC analogue group (*n* = 52) [21]. The primary efficacy parameters were stool weight, abnormal stool frequency and score of diarrhea questionnaire. All the patients have no recognized enteritis or intestinal canal identified from enteroscope or diarrhea resulted by protease inhibitors (PI) drugs. According to an analysis of the treatment effect over 7 and 14 days based on daily measurements, Patients who were treated with XC experienced a statistically significant reduction in stool weight (*P* = 0.0029 in 7 days and *P* = 0.0023 in 14 days) and in diarrhea questionnaire score (*P* < 0.01 in 14 days). There were no significant differences between groups with respect to stool frequency. No serious adverse events were reported. There was no major difference between XC and placebo in the occurrence of adverse events or in laboratory abnormalities.

#### 3.3.10. Aikang Capsule

A randomized placebo controlled trial enrolled 102 patients infected with HIV and AIDS with CD4+ cell counts between 250 and 600 cells/mm^3^ who were treated with Aikang Capsule (Table 1) or placebo for 6 months [22]. There was no significant difference in CD4+ cell counts between two groups.

#### 3.3.11. Tangcao Tablets

In a China phase III clinical muti-center trial conducted between 2002 and 2003, 176 patients with CD4+ cell counts >200 cells/mm^3^ were randomized to receive a 6- month course of treatment with Chinese herbal medicine Tangcao Tablets (Table 1) (*n* = 88) or placebo (*n* = 88) [23]. Patients receiving antiretroviral drugs were excluded. Both intention to treat analysis and per-protocol analysis showed significant increase in CD4 counts, CD4/CD8 ratio and weight in herb group, significant increase of viral load in placebo group, improvement of symptoms in herb group.

The total number of reported adverse events was 21 in the herb group and 27 in the placebo group, and included diarrhea, cold, abdominal pain, flatulence, and nausea. Hematological or serum chemistry laboratory values showed no evidence of toxicity from the study herbs. Two patients in the placebo group died during the study period and causes of death were believed to be due to severe immunodeficiency and pre-enrolment history of severe opportunistic complications, but not related to the placebo.

## 4. Discussion

Due to the limited number of RCTs for included trials, the small sample size of each study, we are not able to draw firm conclusions concerning TCHM therapy in treating patients with HIV infection and AIDS. Compared with placebo, TCHM demonstrated positive effects in improving QoL and symptoms, increasing CD4+ cell counts; however, the quality of studies need to be concerned. Our paper aimed to update and complete the evidence of TCHM treatments for patients with HIV infections and AIDS. Compared to a previous paper [10], we identified 6 new RCTs and successfully updated the evidence. The results of our paper are similar to that of the previous paper [10], which also expressed concern regarding the beneficial effects need to be considered with caution because the number of patients in these trials was small and the size of the effects quite moderate.

Some studies support the Chinese herb and antiretroviral drug combination therapy [14, 18]. A promising additional antiviral benefit was found from Compound SH combined with antiretroviral agents; however, high drop out rate should be taken into consideration. Wang Jian et al. provide evidence in improving symptoms and a lower risk for the decrease of CD4+ cell counts for patients with combined therapy using Chinese herb Aining granule. Studies of diarrhea and oral candidiasis, which are challenging symptoms of AIDS, were demonstrated to have positive effects [19, 21]. Study of peripheral leukocytes, which are a side effect of antiretroviral drugs, suggested that an integrated treatment approach may be of benefit [20]. The use of Chinese herbs is associated with non-serious adverse effects in our included trials. However, potential risk of interaction between herbs and antiretroviral drugs need more explorations.

The methodological limitations of the studies reported in this systematic literature review included small sample sizes, non-reporting of followup, insufficient details on sampling, high drop-out rates, lack of intention to treat analysis and lack of blinding. The reporting of studies was also very limited for included papers, with items most commonly missing from the CONSORT checklist including: 1a (identification as RCT in title); 16 (numbers of participants included in each analysis); 6b (changes to trial outcomes); 8, 9, and 10 (details of randomisation procedure); 14b (why the trial was ended); and 23 and 24 (registration number and full protocol access) [25]. STRICTA guidelines should be adopted [26].

Our paper has a number of important limitations. Although strong efforts were made to retrieve all RCTs on the subject, we cannot be absolutely certain that we succeeded. Moreover, selective publishing and reporting are other major causes for bias, which must be considered. It is conceivable that several negative RCTs remain unpublished. Together, these factors limit the conclusiveness of this systematic review considerably.

In conclusion, the results of our systematic review provide limited evidence for the effectiveness of TCHM in treating patients with HIV infection and AIDS. Thus, drawing firm conclusions concerning the effectiveness of CAM therapies remains difficult. Further large and rigorous RCTs are warranted.

## Figures and Tables

**Table 1:** Summary of randomized clinical studies of TCM for treating HIV infections and AIDS.

Study (country)	Design	Participants (*n*)	Treatment	Control	Outcome measures	Main findings
Burack et al. 1996 (US) [13]	Parallel, two arms, Double-blind trial	Symptomatic patients infected with HIV with decreased CD4 cells (30)	Chinese herbal preparation (IGM-1) for 12 weeks	Placebo	Symptoms, CD4 cell counts, qulity of life, adverse effects	Overall life satisfaction improved in patients treated with herbs, no difference in CD4 count and symptom severity

Sangkitporn et al. 2005 (Thailand) [14]	Muticentre, double-blind, placebo-controlled trial	Adults with HIV-1 infection (60)	Chinese herbal compound (SH) plus ZDV and ddC for 24 weeks	Placebo plus ZDV and ddc for 24 weeks	HIV RNA, CD4 counts, adverse effects	Significant decrease in HIV RNA levels in SH group than placebo without serious adverse events

Shi and Peng 2003 (China) [15]	Parallel, two arms, Double-blind trial	Adult patients infected with HIV and AIDS (36)	Qiankunning (extracts from 14 herbs) for 7 months	Placebo	CD4 cell counts, viral loads, adverse effects	Significant decrease in HIV RNA levels in herb group than placebo. Use of herbs was related to gastroenterological adverse effects.

Wang et al. 2006 (China) [16]	Parallel, double-blind, placebo-controlled trial	Patients infected with HIV and AIDS (72)	Chinese herbal preparation ZY-4 for 6 months	Placebo	CD4 cell counts, viral loads, symptom, body weight, adverse effects	Significant increase of CD4 counts in ZY-4, but not significant difference on symptoms, weight or viral load between groups

Weber et al. 1999 (Switzerland) [17]	Parallel, two arms, Double-blind trial	Adults infected with HIV with decreased CD4 cells (68)	Chinese herbs (35 herbs) for 6 months	Placebo	AIDS event, CD4 cell counts, viral load, quality of life, adverse effects	No positive findings for the outcome and herbs associated with adverse effects

Wang et al. 2008 (China) [18]	Parallel, two arms, placebo-controlled Double-blind trial	adults infected with HIV, received HAART therapy for 0.5–1 year (100)	Chinese herbal preparation Aining Granule (AG) plus d4T, ddI and NVP for 11 months	placebo plus d4T, ddI and NVP for 11 months	Symptoms, CD4 cell counts, viral loads, CD8, IL-2, IL-4, IFN-γ, adverse effects	Significant decrease of CD4 counts in placebo group, improvement of symptoms of anepithymia, diarrhea and nausea, but not significant difference on viral load, CD8, IL-2,4 between groups

Jiang et al. 2009 (China) [19]	Parallel, two arms, controlled open label trial	Patients who are HIV infection and AIDS with oral candidiasis symptoms (80)	Chinese herbal preparation XiaoMi Granule (XMG) plus Nystatin for external use for 2 weeks	Nystatin for 2 weeks	Symptoms of oral candidiasis, adverse effects	Significant improvement of symptoms of oral candidiasis in herb group, no adverse event was found

Jiang et al. 2011 (China) [20]	Parallel, double-blind, double dummy trial	Patients who are HIV infection and AIDS with leukopenia symptoms (116)	Chinese herbal preparation Jingyuankang Capsule (JC) plus AZT, ddI, NVP and analogue Leucogen Tablets for 6 months	Leucogen Tablets plus AZT, ddI, NVP and analogue JC	Peripheral leukocytes, adverse effects	Significant increase of peripheral leukocytes without serious adverse events

Xu et al. 2011 (China) [21]	Parallel, double-blind, double dummy trial	Patients with AIDS and diarrhea (158)	Chinese herbal preparation Xielikang Capsule (XC) plus analogue loperamide for 14 days	Loperamide plus analogue XC for 14 days	Symptoms of diarrhea, adverse effects	Improvement of symptom of diarrhea in herb group

Xie et al. 2008 (China) [22]	Parallel, two arms, placebo-controlled Single-blind trial	Patients infected with HIV and AIDS with CD4 250–600 cells/mm^3^ without HAART therapy (102)	Aikang Capsule (AC) for 6 months	Placebo for 6 months	CD4 cell counts	No significant difference between groups.

Shao 2008 (China) [23]	Parallel, two arms, placebo-controlled Double-blind trial	Patients infected with HIV and AIDS Without HAART therapy (176)	Tangcao Tablets (TT) for 6 months	Placebo for 6 months	CD4 cell counts, viral loads, symptom, body weight, adverse effects	Significant increase of CD4 counts, CD4/CD8 and weight in herb group, significant decrease of viral load in placebo group, improvement of symptoms in herb group.

**Table 2:** Risk of bias of included RCTs*.

Study	Random sequence generation	Allocation concealment	Patient blinding	Assessor blinding	Reporting drop-out or withdrawal^†^	Intention-to-treat analysis^†^	Selective outcome reporting	Other potential bias
Wang et al. 2010 [7]	Low	Low	Low	Low	Low	Low	Low	Low
Hsiao et al. 2003 [8]	Unclear	Low	Low	Low	Low	High	Low	Unclear
Özsoy and Ernst 1999 [9]	Low	Low	Low	Low	Low	High	Low	Low
Liu et al. 2005 [10]	Low	Low	Low	Low	Low	High	Low	Low
Bishop et al. 2007 [11]	Low	Low	Low	Low	Low	Low	Low	Low
Tsao et al. 2005 [12]	Low	Low	Low	Low	Low	High	Low	Low
Higgins et al. 2011 [24]	Unclear	Low	Low	Low	Low	Low	Low	Unclear
Burack et al. 1996 [13]	Low	High	High	High	Low	Low	Low	Unclear
Sangkitporn et al. 2005 [14]	Low	Low	Low	Low	Low	Low	Low	Low
Shi and Peng 2003 [15]	Low	Low	Low	High	Low	Low	Low	Unclear
Wang et al. 2006 [16]	Low	Low	Low	Low	Low	Low	Low	Low

*Domains of quality assessment based on Cochrane tools for assessing risk of bias.

^†^Two domains referring to “incomplete outcome data” in the Cochrane tools for assessing risk of bias.

Abbreviations: low: low risk of bias; high: high risk of bias; unclear: uncertain risk of bias.
